# Immunomodulatory Effect of MSC on B Cells Is Independent of Secreted Extracellular Vesicles

**DOI:** 10.3389/fimmu.2019.01288

**Published:** 2019-06-06

**Authors:** Laura Carreras-Planella, Marta Monguió-Tortajada, Francesc Enric Borràs, Marcella Franquesa

**Affiliations:** ^1^REMAR-IVECAT Group, Germans Trias i Pujol Health Science Research Institute, Badalona, Spain; ^2^Department of Cell Biology, Physiology and Immunology, Autonomous University of Barcelona, Barcelona, Spain; ^3^ICREC Research Program, Germans Trias i Pujol Health Science Research Institute, Badalona, Spain; ^4^Nephrology Service, Germans Trias i Pujol University Hospital, Badalona, Spain

**Keywords:** mesenchymal stromal cells, exosome, regulatory B cell, immunosuppression, memory B cell, Ev isolation

## Abstract

Mesenchymal stem or stromal cells (MSC) have proven immunomodulatory properties toward B cell activation and induce regulatory B cells (Breg), through a dual mechanism of action that relies both on cell contact and secreted factors. One of them are MSC-derived extracellular vesicles (EVs), membrane nanovesicles that mediate cell communication and typically reflect the phenotype of the cell of origin. MSC-EVs could resemble MSC functions, and are being contemplated as an improved alternative to the MSC-based immunomodulatory therapy. In the present work, we focused on the factors secreted by MSC and aimed to elucidate the putative role of MSC-EVs in the immunomodulation of B cells. EVs and soluble protein-enriched fractions (PF) were isolated from MSC-conditioned medium (CM) using size-exclusion chromatography (SEC) and their capacity to modulate B cell activation, induction of Breg and B cell proliferation was compared to that of the whole MSCs. Co-culture with MSC or unfractionated CM induced naïve and CD24^hi^CD38^hi^, IL-10 producing (Breg) phenotypes on B cells while not affecting proliferation. MSC-PF had a comparable effect to MSCs, inducing a naïve phenotype, and even though they did not induce the shift toward a CD24^hi^CD38^hi^ population, MSC-PF fostered IL-10 production by B cells. Conversely, MSC-EVs failed to promote naïve B cells and to reduce memory B cells. MSC-EVs induced CD24^hi^CD38^hi^ B cells to a similar extent of that of MSC, but not *bona fide* Bregs since they did not produce IL-10. Our results show that B cell modulation by MSC is partially mediated by soluble factors other than EVs.

## Introduction

Mesenchymal stem or stromal (MSC) are immunomodulatory toward numerous immune cell types *in vitro* as well as *in vivo* (1–3). We recently showed their ability to induce regulatory (Breg) and naïve B cells while reducing activated and memory B cells (4). While the exact mechanism of action remains unclear (5), both cell-contact and secreted factors are needed for MSC modulation of B cells (6, 7). Some cytokines and growth factors have been identified as key mediators amid secreted factors, but more recently the focus has been put on extracellular vesicles (EVs). EVs are membrane nanovesicles that carry molecules reflecting the phenotype and functions of the cells of origin (8). MSC-derived EVs have been shown to emulate their effect on B cells and other immune cells (9–11). However, parameters related to the EV isolation method -including purity- are key to downstream analyses. Widely used techniques such as ultracentrifugation (UC) or precipitating agents-based methods cause the co-precipitation of EVs with other potentially confusing soluble molecules (12), whilst size-exclusion chromatography (SEC) is being considered the method of choice to highly enrich functional EVs (13).

The purpose of the present study is to use SEC to dissect the role of MSC-EV from secreted soluble factors in order to deepen in the mechanisms of B cell immunomodulation by MSC.

## Materials and Methods

### Mesenchymal Stem or Stromal Cell Isolation and Cell Culture

Subcutaneous adipose tissue was obtained from patients undergoing heart surgery in University Hospital Germans Trias i Pujol (HUGTiP). Informed consent was obtained from all subjects, and the study protocol conformed to the principles outlined in the Declaration of Helsinki. Mesenchymal stem or stromal cells (MSC) were isolated from fat tissue as previously described (4, 14). MSC, which were used in passages between 3 and 10, were cultured in αMEM (Sigma Aldrich) supplemented with 10% FBS (Lonza), penicillin (100 IU/ml, Cepa S.L., Madrid, Spain), streptomycin (100 mg/ml, Normon Laboratories S.A., Madrid, Spain) and 2 mM L-Glutamine (Sigma Aldrich).

### Preparation of Conditioned Medium

Two million MSC were seeded in cell culture flasks with 15 ml of complete medium depleted from fetal bovine serum (FBS)-derived EVs (11). To deplete medium from FBS-EVs, 20% FBS complete medium (αMEM +1% P/S +2 mM L-Glutamine) was ultracentrifuged at 100,000 × *g* for 16 h in polypropylene ultracentrifugation tubes (Beckman coulter, Brea, CA). The supernatant was collected and filtered through a 0.22 μm filter (Sarstedt, Germany) to sterilize the medium, which was finally diluted with αMEM medium to the final concentration of 10% FBS for cell culture. After 48 h, the medium was collected and centrifuged at 400 × *g* and 2,000 × *g* to eliminate cells and cell debris, respectively, to obtain MSC-conditioned medium (CM).

### Extracellular Vesicles and Soluble Protein Separation and Analysis

#### Size-Exclusion Chromatography

**Figure 1:**
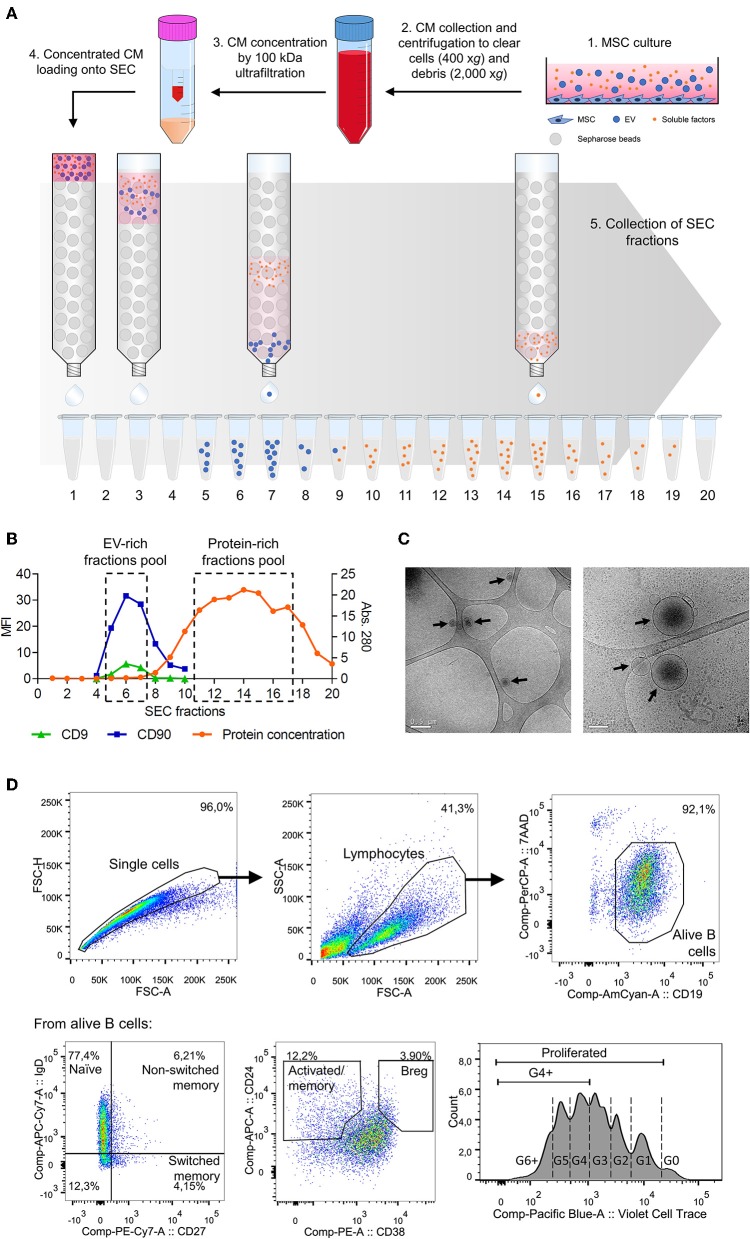
**(A)** Workflow of the methodology used to isolate MSC-EV and MSC-PF by SEC from MSC-CM. (1) Supernatant was collected after 48 h of MSC culture and (2) sequentially centrifuged at 400 × *g* for 5 min and at 2,000 × *g* for 10 min to exclude cells and cell debris, respectively. The obtained MSC-CM was partially kept for experimental use in B cell culture and the rest was then (3) concentrated by 100 kDa ultrafiltration. (4) Concentrated MSC-CM was loaded into a 1 ml SEC sepharose column and (5) 100 μl fractions (up to 20) were collected immediately after loading. **(B)** Representative plot showing the SEC elution profile according to protein concentration and median fluorescence intensity (MFI) of EV markers of each SEC fraction. Fractions with the highest CD9 and CD90 MFI or were pooled together, similarly to fractions with the highest protein concentration, to obtain the MSC-EV or MSC-PF preparations, respectively. **(C)** Cryo-TEM was used to analyze MSC-EV size and morphology. Black arrows point EV in the preparations over the TEM grid. Scale bars = 0.5 μm (left) and 0.2 μm (right). **(D)** Gating strategy followed to define B cell subsets by flow cytometry. Doublets and debris were excluded by FSC-A/FSC-H and FSC-A/SSC-A, respectively, by gating on singlets and lymphocyte populations. Alive B cells were further gated as CD19^+^7AAD^−^, from which each subset was defined: naïve (CD27^−^IgD^+^), non-switched memory (CD27^+^IgD^+^), switched memory (CD27^+^IgD^−^), activated/memory (CD24^hi^CD38^int/lo^), and Breg (CD24^hi^CD38^hi^). Bottom right panel shows a representative proliferation plot where cell generations are indicated with dashed lines. For analysis, the percentages of total proliferated cells (G1 onwards) and G4+ proliferated cells (G4 onwards) were calculated.

MSC-CM was concentrated using a 100 kDa ultrafiltration unit (Amicon Ultra, Millipore, Millerica MA) and fractioned by SEC using columns of 1 ml sepharose CL-2B (Sigma Aldrich). Figure 1A schematically depicts the followed protocol, which can be read in detail in Monguió-Tortajada et al. (15).

#### Bead-Based Flow Cytometry

The EV-enriched SEC fractions were determined by beads-based flow cytometry according to the presence of CD9 and CD90 following the previously described procedure (11, 15) (Figure 1B). Briefly, EVs were coupled to 4 μm aldehyde/sulfate latex microspheres and were then labeled with the fluorochrome-conjugated antibodies anti-CD90-PE-Cy7 or indirectly labeled with the primary antibodies anti-CD9 (Clone VJ1/20) and or the IgG isotype control and secondary antibody FITC-conjugated Goat F(ab')2 Anti-Mouse IgG. The MSC-EV fractions with the highest CD9 and CD90 MFI were pooled to obtain highly enriched MSC-EV preparations.

#### Cryo-Transmission Electron Microscopy (Cryo-TEM)

The presence of EVs in EV-enriched fractions was confirmed by cryo-transmission electron microscopy (cryo-TEM) as previously described (16).

#### Protein Quantification

Protein elution was checked by reading the absorbance (Abs.) at 280 nm of each fraction using Nanodrop® ND-1000 (Thermo Scientific) to pool fractions with the highest protein concentration and obtain MSC-PF preparations.

### B Cells

#### Isolation From Tonsils

Tonsils were obtained from children undergoing routine tonsillectomy after the informed consent of their legal tutors (HUGTiP). The study protocol followed the principles of the Declaration of Helsinki. To obtain B cells, tonsils were mechanically disaggregated with a scalpel, washed with PBS, mononuclear cells were isolated by ficoll differential centrifugation (GE Healthcare) and frozen (liquid N_2_). For the experiments, tonsil cells were thawed and negatively sorted (MACS, Miltenyi Biotech) to obtain the CD43^−^ population (mature inactivated B cells).

#### Activation and Culture

One hundred-thousand B cells were seeded in flat bottom 96 well plates, T-cell-like-stimulated with IL-2/anti-IgM/anti-CD40 and co-cultured with 10,000 MSC for 7 days in a 10:1 B:MSC ratio as previously described (4, 7).

B cells were also cultured with MSC-CM, MSC-EV, or MSC-PF. Of each condition, 100 μl of MSC-EV or MSC-PF were added to the culture. This volume corresponded to EVs and PF secreted by 50,000 MSC which is 5 times the amount of MSC added in the B cell-MSC co-culture. In the dose-dependency experiments, half (50 μl) or one tenth (10 μl) of the initial volume of CM or PF conditions were used.

#### B Cell Subsets and Proliferation Assessment by Flow Cytometry

After 7 days, B cells were collected and processed for flow cytometric analysis to analyze B cell populations (FACS Canto II, BD Biosciences) as previously described (4, 7). Viability was assessed by 7AAD staining (BD Biosciences) and quantified as CD19^+^7AAD^−^ cells among the lymphocyte population. In separate experiments, B cells were labeled with Violet CellTrace (Invitrogen) for 20 min at 37°C to analyze proliferation by dye dilution. Figure 1D shows the gating strategy followed.

### IL-10 and TNFα Quantification by ELISA

After co-culture, supernatants were harvested and stored at −80°C for later IL-10 and TNFα quantification using dedicated ELISA kits (U-CyTech).

### Data Analysis

All the experiments were performed with an allogenic co-culture of MSC and B cells from eleven and five different donors, respectively. Technical duplicates were used. Flow cytometry data was analyzed by FlowJo v10 software (FlowJo LLC, Ashland, OR). Statistical analysis was performed with Prism v6 software (GraphPad, La Jolla, CA). Kruskall-Wallis with Dunn's multi-comparison test was used to determine statistical differences between groups (*p* < 0.05 was considered significant).

## Results

### EV Isolation and Analysis

MSC-CM obtained after 48 h of culture was collected concentrated and fractionated by SEC. Fractions eluted from SEC were evaluated for CD9 and CD90, which are known EV (8) and MSC-EV markers (17), respectively (Figure 1A). As previously described by our group (11), we consistently observed that SEC EV-enriched fractions were free from the bulk of soluble proteins, that eluted in later fractions. MSC-EV were further characterized by Cryo-TEM, confirming the presence of nanosized EV (Figure 1C).

### B Cell Survival Relies on Cell-To-Cell Contact

**Figure 2:**
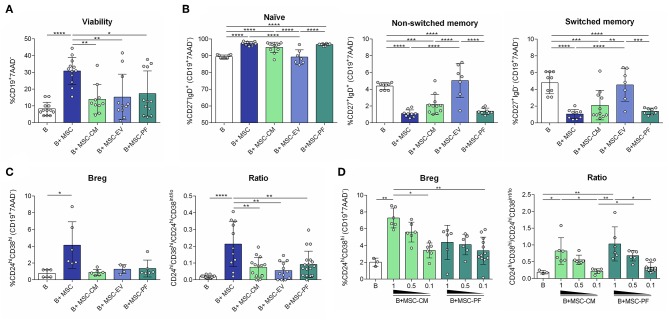
Flow cytometry was used to measure **(A)** percentages of alive B cell (CD19^+^7AAD^−^); **(B)** percentages of B cell subsets: naïve (CD27^−^IgD^+^), non-switched memory (CD27^+^IgD^+^), switched memory (CD27^+^IgD^−^); **(C)** the percentage of Breg (CD24^hi^CD38^hi^) and the ratio between Breg (CD24^hi^CD38^hi^) and activated/memory (CD24^hi^CD38^int/lo^) for each condition, and **(D)** in a dose-dependency experiment for PF and CM conditions. Each dot represents a different combination of MSC and B cells donors. Statistical significance (*p* < 0.05) was determined by Kruskall-Wallis with Dunn's multi-comparison test (^*^p ≤ 0.05, ^**^p ≤ 0.01, ^***^p ≤ 0.001, ^****^p ≤ 0.0001). MSC, mesenchymal stem cells; CM, conditioned medium; EV, extracellular vesicles; PF, protein fraction.

We have previously shown that MSC increase B cell survival compared to non-stimulated B cells but also compared to T cell-like activated B cells (IL-2/anti-IgM/anti-CD40) (4). In the current setting, the number of living B cells (CD19^+^7AAD^−^) was significantly increased in the co-cultures with MSC but not in the other conditions, compared to stimulated B cells alone (Figure 2A).

### B Cell Plasticity Mediated by MSC Is Independent of MSC-EVs

B cell population analysis by flow cytometry revealed that MSC-CM and MSC-PF retained B cells in a naïve state similarly to MSC contact, and reduced non-switched and switched memory B cells. MSC-EV did not change B cell populations of stimulated B cells (Figure 2B).

### The MSC-PF but Not MSC-EV Is Partially Responsible of Breg Induction

We next assessed the induction of Breg, phenotypically defined here as CD24^hi^CD38^hi^CD19^+^ B cells and by secretion of IL-10 and low secretion of TNFα. The CD24^hi^CD38^hi^ phenotype was only significantly induced by MSC co-culture. Since activated and memory B cells represent the CD24^hi^CD38^int/lo^ subset, we defined the ratio CD24^hi^CD38^hi^/CD24^hi^CD38^int/lo^ as an index between transitional immunosuppressive (CD24^hi^CD38^hi^) and inflammatory activated/memory (CD24^hi^CD38^int/lo^) B cell populations. In this case, MSC significantly generated a higher immunoregulatory ratio than any of the other conditions (Figure 2C).

To further confirm that the paracrine effect of MSC on Breg polarization is mediated by the PF of the CM, a dose-dependency experiment was performed. We observed that the percentage of CD24^hi^CD38^hi^ cells and the ratio CD24^hi^CD38^hi^/CD24^hi^CD38^int/lo^ were higher when the highest dose of PF and CM was added to cells, showing that this effect is related to the CM and its soluble protein-enriched fractions (Figure 2D). Moreover, the PF as well as the CM could increase the percentage of non-switched and switched memory subsets in a dose-dependent manner (data not shown).

**Figure 3:**
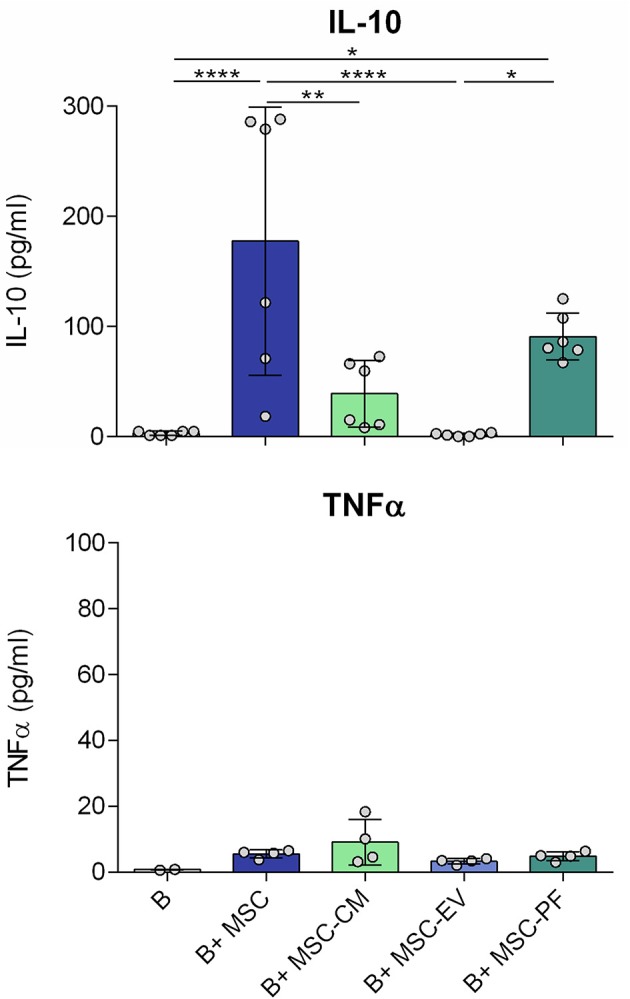
Concentration of IL-10 and TNFα in the culture medium after 7 days of B cell culturing with the different conditions measured by ELISA. Each dot represents a different combination of MSC and B cells donors. Statistical significance (*p* < 0.05) was determined by Kruskall-Wallis with Dunn's multi-comparison test (^*^p ≤ 0.05, ^**^p ≤ 0.01, ^****^p ≤ 0.0001). MSC, mesenchymal stem cells; CM, conditioned medium; EV, extracellular vesicles; PF, protein fraction.

In line, the IL-10 concentration of MSC co-culture condition was also significantly higher than in any other condition, except for MSC-PF. MSC-PF significantly induced more IL-10 secretion than MSC-EV and B cells alone. Noticeably, TNFα was secreted at very low concentrations in all conditions (< 20 pg/ml) (Figure 3).

### Assessment of B Cell Subsets Proliferation

**Figure 4:**
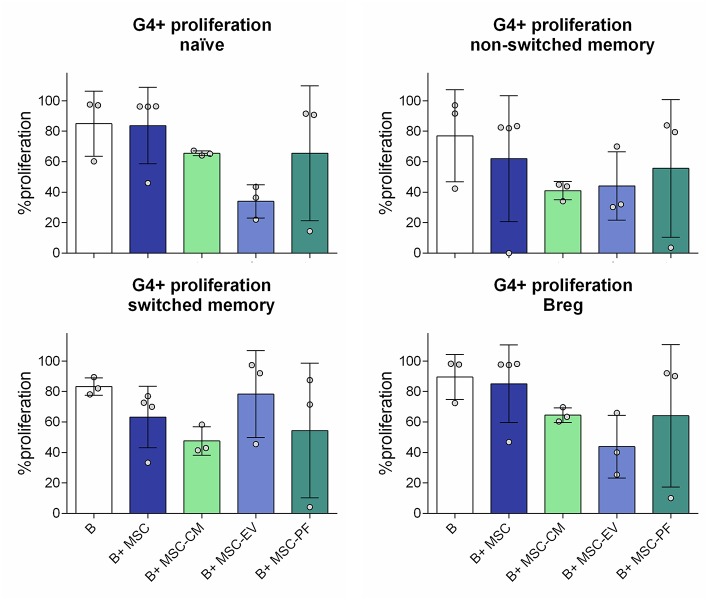
Percentage of proliferation from the 4th generation onwards (G4+ proliferation) of the different B cell subsets measured by dilution of Violet CellTrace dye by flow cytometry. The different subsets were gated from alive B cells (CD19^+^7AAD^−^). Naïve, CD19^+^CD27^−^IgD^+^; non-switched memory, CD27^+^IgD^+^; switched memory, CD27^+^IgD^−^; Breg, CD24^hi^CD38^hi^. No statistically significant differences were found between conditions by Kruskall-Wallis with Dunn's multi-comparison test.

We also compared the effect of whole MSC with their secreted factors in terms of B cell subsets proliferation. In our setting, after 7 days >80% of B cells proliferated in all conditions with few changes between groups (data not shown), so we focused on the study of 4th generation onwards proliferation (G4+). MSC co-culture allowed B cell proliferation G4 onwards whilst MSC-CM and its derived fractions did not reach the same percentage, even though the differences did not reach statistical significance. We further observed a trend of MSC-EV compared MSC-PF to reduce proliferation of naïve, non-switched memory B cells and Bregs while increasing switched memory proliferation (Figure 4).

## Discussion

In the present work we studied the role of the MSC secreted factors on B cell immunomodulation. We isolated EVs by SEC, which allows to obtain purer EV preparation to properly discriminate it from the non-EV soluble fraction.

Ultracentrifugation (UC) without any extra purification step has been the solely and most widely used method for EV isolation, as reflected in the majority of published articles. However, UC is not an appropriate method to isolate EV since it affects the biophysical properties and co-precipitate EV with other particles (proteins, membrane fractions…) that are interfering with the subsequent molecular or functional analyses (18–20). Recently, more refined techniques for EV purification have made their way into the field which are slowly changing the paradigm about the properties and functional characteristics of EV. Within these methods, SEC stands out for its better preservation, higher yield and purity, and ease for EV isolation (12, 21). Several groups have assessed the differences between UC and SEC for downstream analyses, concluding that UC co-precipitate EVs and soluble proteins hampering the discrimination of their individual effect in functional studies (11, 13, 22, 23).

Conditioned medium (MSC-CM) was fractionated by SEC to obtain EV-enriched and non-EV soluble protein fractions, and their distinct effect on B cell subsets and proliferation after T-cell like activation was analyzed. MSC-CM and MSC-PF had a comparable immunomodulatory effect to that of MSC on B cells—increasing the proportion of naïve B cells and IL-10 production and reducing memory B cell phenotype proportions. In contrast, MSC-EV did not exert any effect on activated B cells. Our results contrast with those obtained before that postulate MSC-EV as important mediators for B cell immunomodulation (9, 24). One of the key points of this difference is the method used to isolate and purify the EVs.

The effect of MSC-PF is similar but to a lower level than MSC co-culture. This can be due to the fact that the modulation of B cells by MSC is partially cell-contact dependent, but it also could be explained by differences in B cells' stimulation time frame, since factors are secreted continuously throughout 7 days in the co-culture. To counterbalance this effect, and because a well-defined quantification of EVs is missing in the field, the amount of MSC-EV and MSC-PF used was coming from a proportionally higher number of cells than those used in the co-culture setting. This approach avoids the potential underestimation of the effect of EV or soluble factors due to an insufficient amount.

The lack of unique markers for Breg complicates their analysis. While the transitional phenotype CD24^hi^CD38^hi^ appears as one of the most accepted, IL-10 production is still the key feature to define Breg. In our hands, the transitional phenotype did not fully correlate with IL-10 secretion, since the percentage of CD24^hi^CD38^hi^ was only increased in the MSC co-culture setting compared to B cells alone, while IL-10 concentration was significantly increased in both the co-culture as well as with MSC-PF. However, we experimentally observed that the ratio transitional (CD24^hi^CD38^hi^)/activated-memory B cells (CD24^hi^CD38^int/lo^) correlated better with IL-10 production. This argues for the hypothesis that a favorable balance between immunomodulatory and pro-inflammatory phenotypes is needed for immunomodulation, rather than fostering immunomodulatory phenotypes alone.

Proliferation experiments rendered a similar percentage of proliferating cells in B cells alone and MSC conditions. However, as the viability of B cells alone was significantly lower, the total number of proliferated B cells was higher in the MSC co-culture. Results were not significantly different between MSC-CM or its derived fractions, but the mean proliferation percentage was lower than in the co-culture, suggesting a cell-contact based mechanism to fulfill an optimal MSC conditioning. Still, we observed a trend of MSC-CM and MSC-PF to better reflect the effect of MSC co-culture compared to MSC-EV supporting the idea that MSC-PF better reflects the immunomodulatory effect of MSC.

Using SEC as one of the most refined current techniques to separate CM's EV and soluble proteins, we can conclude that the partial effect of MSC soluble factors on B cell immunomodulation is preferentially induced not by EV but rather by the protein-enriched fractions. The actual factors responsible of the effect are still under investigation.

## Ethics Statement

This study was carried out in accordance with the recommendations of Guideline for Good Clinical Practice from the Comitè d'Ètica de la investigació clínica de l'Hospital Universitari Germans Trias i Pujol with written informed consent from all subjects. All subjects gave written informed consent in accordance with the Declaration of Helsinki. The protocol was approved by the Comitè d'Ètica de la investigació clínica de'Hospital Universitari Germans Trias i Pujol.

## Author Contributions

LC-P contributed to collection of data analysis and interpretation and manuscript writing. MM-T and FB contributed to data analysis and interpretation, and final approval of manuscript. MF conception and design, collection of data, data analysis and interpretation, and manuscript writing.

### Conflict of Interest Statement

The authors declare that the research was conducted in the absence of any commercial or financial relationships that could be construed as a potential conflict of interest.
