# Corrigendum: Immunomodulatory Effect of MSC on B Cells Is Independent of Secreted Extracellular Vesicles

**DOI:** 10.3389/fimmu.2019.02413

**Published:** 2019-10-15

**Authors:** Laura Carreras-Planella, Marta Monguió-Tortajada, Francesc Enric Borràs, Marcella Franquesa

**Affiliations:** ^1^REMAR-IVECAT Group, Germans Trias i Pujol Health Science Research Institute, Badalona, Spain; ^2^Department of Cell Biology, Physiology and Immunology, Autonomous University of Barcelona, Barcelona, Spain; ^3^ICREC Research Program, Germans Trias i Pujol Health Science Research Institute, Badalona, Spain; ^4^Nephrology Service, Germans Trias i Pujol University Hospital, Badalona, Spain

**Keywords:** mesenchymal stromal cells, exosome, regulatory B cell, immunosuppression, memory B cell, Ev isolation

There is an error in the Funding statement detailed in the ***Acknowledgments*** section. The correct phrasing and number for “Instituto Carlos III (PI17/00336)” is “project PI17/00335, integrated in the National R+ D+ I and funded by the ISCIII and the European Regional Development Fund.”

A correction has been made to the ***Acknowledgments*** section:

“This work was supported in part by Fundació La Marató de TV3 (201516-10, 201502-30), SGR programme of Generalitat de Catalunya (2017-SGR-301 REMAR Group), ISCIII-REDinREN (RD16/0009 Feder Funds) and Instituto Carlos III project PI17/00335, integrated in the National R+ D+ I and funded by the ISCIII and the European Regional Development Fund. LC-P by the Spanish Government FPU grant (Formación de Personal Universitario, FPU17/01444); MM-T is sponsored by the PERIS (SLT002/16/00234) from the Generalitat de Catalunya; FB is a researcher from Fundació Institut de Recerca en Ciències de la Salut Germans Trias i Pujol, supported by the Health Department of the Catalan Government (Direcció General de Recerca i Innovació, Department Salut, Generalitat de Catalunya) and MF is funded by the Catalan Health Department (Generalitat de Catalunya) contract PERIS (SLT002/16/00069); the authors also want to thank Miriam Morón-Font for the graphical art help.”

Additionally, in the original article, there was a mistake in Figure 3 as published. In the X axis of the figure, instead of B+ MSC-FP, it should be B+ MSC-PF. The corrected Figure 3 appears below. The authors apologize for these errors and state that they do not change the scientific conclusions of the article in any way. The original article has been updated.

**Figure 3:**
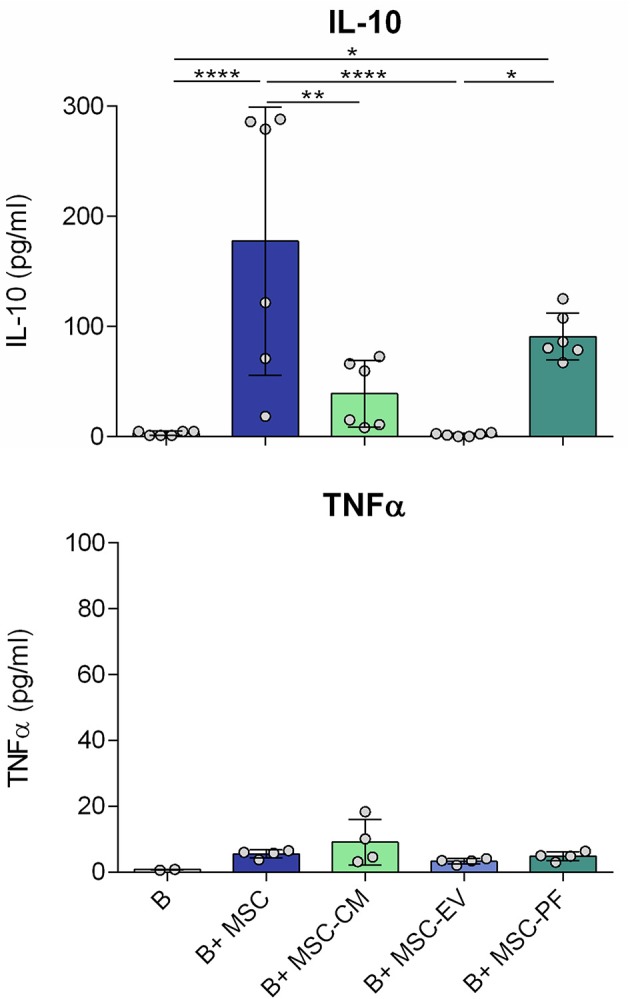
Concentration of IL-10 and TNFα in the culture medium after 7 days of B cell culturing with the different conditions measured by ELISA. Each dot represents a different combination of MSC and B cells donors. Statistical significance (*p* < 0.05) was determined by Kruskall-Wallis with Dunn's multi-comparison test (^*^p ≤ 0.05, ^**^p ≤ 0.01, ^****^p ≤ 0.0001). MSC, mesenchymal stem cells; CM, conditioned medium; EV, extracellular vesicles; PF, protein fraction.

